# Attitudes about use of preoperative risk assessment tools: a survey of surgeons and surgical residents in an academic health system

**DOI:** 10.1186/s13037-022-00320-1

**Published:** 2022-03-17

**Authors:** Nisha Pradhan, Adam R. Dyas, Michael R. Bronsert, Anne Lambert-Kerzner, William G. Henderson, Howe Qiu, Kathryn L. Colborn, Nicholas J. Mason, Robert A. Meguid

**Affiliations:** 1 Surgical Outcomes and Applied Research Program, Department of Surgery, University of Colorado School of Medicine, Aurora, CO USA; 2 Division of Cardiothoracic Surgery, Department of Surgery, Anschutz Medical Campus, University of Colorado Denver, 12631 E. 17th Avenue, C-310, Aurora, CO 80045 USA; 3 Adult and Child Center for Health Outcomes Research and Delivery Science, University of Colorado School of Medicine, Aurora, CO USA; 4 Department of Biostatistics and Informatics, Colorado School of Public Health, Aurora, CO USA

**Keywords:** Risk assessment, SURPAS, NSQIP, VASQIP

## Abstract

**Background:**

Formal surgical risk assessment tools have been developed to predict risk of adverse postoperative patient outcomes. Such tools accurately predict common postoperative complications, inform patients and providers of likely perioperative outcomes, guide decision making, and improve patient care. However, these are underutilized. We studied the attitudes towards and techniques of how surgeons preoperatively assess risk.

**Methods:**

Surgeons at a large academic tertiary referral hospital and affiliate community hospitals were emailed a 16-question survey via REDCap (Research Electronic Data Capture) between 8/2019-6/2020. Reminder emails were sent once weekly for three weeks. All completed surveys by surgical residents and attendings were included; incomplete surveys were excluded. Surveys were analyzed using descriptive statistics (frequency distributions and percentages for categorical variables, means, and standard deviations for continuous variables), and Fisher’s exact test and unpaired t-tests comparing responses by surgical attendings vs. residents.

**Results:**

A total of 108 surgical faculty, 95 surgical residents, and 58 affiliate surgeons were emailed the survey. Overall response rates were 50.0% for faculty surgeons, 47.4% for residents, and 36.2% for affiliate surgeons. Only 20.8% of surgeons used risk calculators most or all of the time. Attending surgeons were more likely to use prior experience and current literature while residents used risk calculators more frequently. Risk assessment tools were more likely to be used when predicting major complications and death in older patients with significant risk factors. Greatest barriers for use of risk assessment tools included time, inaccessibility, and trust in accuracy.

**Conclusions:**

A small percentage of surgeons use surgical risk calculators as part of their routine practice. Time, inaccessibility, and trust in accuracy were the most significant barriers to use.

**Supplementary Information:**

The online version contains supplementary material available at 10.1186/s13037-022-00320-1.

## Background

Accurate preoperative assessment of a patient’s surgical risk for postoperative complications is a challenging but important task for surgeons. Although surgical risk assessment tools such as the American College of Surgeons’ Surgical Risk Calculator, [1] the Veterans Affairs Surgical Quality Improvement Program (VASQIP) Patient Risk Calculator, and the Surgical Risk Preoperative Assessment System (SURPAS) [2] accurately predict risk of adverse postoperative patient outcomes, these tools have not been widely adopted by surgeons to routinely assess preoperative surgical risk [3, 4]. Surgeons are accurate at predicting preoperative risk in low-risk patients, but they are inaccurate when predicting risk of adverse events in high-risk patients. [2, 5] This is problematic as high-risk patients are the most likely to benefit from pre-operative counseling, informed consent discussions, and potential risk mitigation efforts.

Implementation of formal preoperative risk assessment may optimize understanding of risk of adverse postoperative outcomes by both surgical teams and patients [3, 4]. Patients desire to understand their risks, and report increased shared decision making with use of such tools [6–9]. The use of personalized risk assessment increases the patient’s understanding of the proposed operation and improves the communication of informed consent and individualized patient risks [10], which improves patient anxiety, comfort about the operation, and satisfaction. Surgeon use of such tools has been shown to lead to more accurate judgments of operative risk [11, 12]. These tools have allowed the operative decision-making process to become more objective in evaluating for potential postoperative complications [13]. Therefore, increased understanding of surgeons’ attitudes about use of risk assessment tools should be useful for further development of these tools.

The purpose of this study was to collect information on surgeons’ attitudes toward surgical risk tools, including how often they are used compared to other methods of risk assessment, how risk is communicated to patients, what factors increase the likelihood of using surgical risk tools, what are barriers to use of surgical risk tools, and which features enhance usefulness of surgical risk tools. It is also important to determine if level of training (e.g., resident vs. attending surgeon) impacts attitudes about surgical risk assessment. We hypothesized that: (1) a small percentage of surgeons routinely use preoperative risk assessment tools; (2) that attending surgeons are more likely to rely on prior experience to guide risk assessment, and residents are more likely to use surgical risk tools; and (3) that time is a significant barrier to use.

## Methods

This research was conducted after review and approval from the Colorado Multiple Institutional Review Board (COMIRB#: 19-1068).

This study was a prospective survey developed by the research team under the guidance of a qualitative specialist with extensive experience in survey design (co-author ALK). The team designed a 16-question multiple choice survey to elicit opinions of and attitudes towards use of formalized risk assessment tools (Fig. S1). We used a series of multiple-choice questions that assessed the respondent’s frequency of risk discussions with patients, methods used, time spent, the sources of the risk information, useful features of a risk assessment tool, and barriers to use. The survey was designed such that respondents could choose to remain anonymous.

The survey was administered to surgical faculty and residents in the Department of Surgery at the University of Colorado Hospital (UCH), the Rocky Mountain Regional Veterans Administration Medical Center, Children’s Hospital of Colorado, Denver Health Hospital, and to affiliate appointments of the Department of Surgery in the years 2019-2020. We included surgical faculty and residents within the surgical specialties of general, trauma/ acute care, surgical critical care, surgical oncology, vascular, cardiac, thoracic, plastic reconstructive, colorectal, transplant, endocrine and urology. Affiliates included academic-affiliate hospitals within the UCHealth system. Survey response was incentivized through two $50 gift card raffles, one for surgical faculty and one for surgical residents. Reminder emails were sent once weekly for three weeks after the initial survey was disseminated. Incomplete survey responses were excluded. Surveys were disseminated and responses collected through REDCap (Research Electronic Data Capture, Vanderbilt University, Nashville, TN), a software tool for designing and collecting data for a research database. In addition to the 16 survey questions, we also collected the following data about the surgeon responders: age, gender, years of practice, specialty, education, hospital type, and hospital bed size.

Descriptive statistics, including frequency distributions and percentages for categorical variables, means, and standard deviations for continuous variables, were used to summarize the survey results. Group comparisons were performed using Fisher’s exact test for categorical variables, and unpaired t-test or Wilcoxon rank sum test for continuous variables. Results were considered statistically significant if the p-value of the test was ≤0.05. All statistical analyses were performed using SAS version 9.4 [SAS Inc, Cary, NC].

## Results

A total of 261 surveys were emailed (108 to faculty surgeons, 95 to residents, and 58 to affiliated surgeons). One hundred twenty (46.0%) were completed (54 faculty surgeons, 45 residents, 21 affiliate surgeons). Response rates were 50.0% for faculty surgeons, 47.4% for residents, and 36.2% for affiliate surgeons. Two additional surveys were received but excluded due to incomplete data or the surgeon no longer being in clinical practice.

Table 1 presents the demographic information of survey participants. Approximately 40% of residents and affiliated surgeons were female, but only 26% of faculty surgeons. Average age and median years of practice of faculty and affiliate surgeons were similar (49.6 and 10 years vs. 46.0 and 12 years, respectively). The 120 surgeons were from general surgery (70.0%), urology (7.5%), vascular (6.7%), cardiac (3.3%), and other specialties (12.5%); and from an academic hospital (UCH) (78.3%), urban community hospital (15.8%), or rural hospital and other settings (5.8%).

**Table 1:** Survey participants’ and hospital characteristics

Characteristics	Total	Affiliated Surgeons	Faculty Surgeons	Resident
	(*n *= 120)	(*n *= 21)	(*n *= 54)	(*n *= 45)
	N (%)*	N (%)*	N (%)*	N (%) *
Female	41 (34.2)	9 (42.9)	14 (25.9)	18 (40.0)
Age, mean (SD)^a^	41.9 (12.3)	46.0 (9.7)	49.6 (11.2)	30.7 (3.0)
Years of practice, years, median (IQR)^a^	5 (1-16)	12 (3-19)	10 (5-20)	0 (0-1)
Surgical specialty				
Cardiac	4 (3.3)	0 (0)	4 (7.4)	0 (0)
General	84 (70.0)	17 (81.0)	23 (42.6)	44 (97.8)
Neurological	1 (0.8)	1 (4.8)	0 (0)	0 (0)
Other	14 (11.7)	1 (4.8)	13 (24.1)	0 (0)
Urologic	9 (7.5)	0 (0)	9 (16.7)	0 (0)
Vascular	8 (6.7)	2 (9.5)	5 (9.3)	1 (2.2)
Education				
MD	97 (80.8)	15 (71.4)	44 (81.5)	38 (84.4)
DO	1 (0.8)	0 (0)	0 (0)	1 (2.2)
MD, MS	8 (6.7)	2 (9.5)	3 (5.6)	3 (6.7)
MD, PhD	4 (3.3)	0 (0)	4 (7.4)	0 (0)
MD, MPH	6 (5.0)	2 (9.5)	2 (3.7)	2 (4.4)
MD, MBA	3 (2.5)	2 (9.5)	0 (0)	1 (2.2)
MD, MHS	1 (1.3)	0 (0)	1 (1.9)	0 (0)
Hospital type				
Academic hospital	94 (78.3)	0 (0)	49 (90.7)	45 (100)
Urban community hospital	19 (15.8)	17 (81.0)	2 (3.7)	0 (0)
Rural hospital	3 (2.5)	3 (14.3)	0 (0)	0 (0)
Other	4 (3.3)	1 (4.8)	3 (5.6)	0 (0)
Bed size				
0-199	13 (10.8)	6 (28.6)	7 (13.0)	0 (0)
200-399	14 (11.7)	9 (42.9)	5 (9.3)	0 (0)
400-599	9 (7.5)	5 (23.8)	3 (5.6)	1 (2.2)
600-799	83 (69.2)	1 (4.8)	38 (70.4)	44 (97.8)
800+	1 (0.8)	0 (0)	1 (1.9)	0 (0)

*Abbreviations*: *SD* standard deviation, *IQR* interquartile range, *MD* Doctor of Medicine, *DO* Doctor of Osteopathic Medicine, *MS* Masters of Science, *PhD* Doctor of Philosophy, *MPH* Master of Public Health, *MBA* Master of Business Administration, *MHS* Master of Health Science.

*Values are frequency and column percent unless otherwise specified

^a^ Variable contained some missing values (Age: *n*=2; Years of practice: *n*=3)

Table 2 presents the 120 respondents’ practices for risk discussion with their patients. The respondents were to record an answer for all categories, and the categories were not mutually exclusive. For comparisons, faculty and affiliate surgeons (i.e., non-trainees) were combined and compared to residents. Among all 120 surgeons, 45.0% spent 5-9 minutes discussing risk, and 36.6% took more than 10 minutes; 72.5% used prior experience (all percentages are most or all of the time, unless otherwise specified) and 60.0% used current literature as their sources of risk estimates, while only 20.8% used online risk calculators as a source of risk estimates. The primary methods (i.e., methods used most or all of the time) that the surgeons used to communicate risk to patients included face to face (90.0%) and pre-anesthesia clinic (51.6%). Phone calls (4.1%), residents (19.2%), advanced practice providers, (4.2%), handouts/pamphlets (19.2%), and classes/videos (4.1%) were used much less frequently. Only 20.8% of the surgeons communicated risk back to the referring provider most or all of the time. Residents spent less time discussing risk with patients (20.0% vs. 46.7% >10 minutes, *p *= 0.004), used risk calculators more often (62.2% vs. 49.4% sometimes to always, *p *= 0.002), and used prior experience (62.2% vs. 78.6%, *p *> 0.0001) and current literature (44.4% vs. 69.3%, *p *= 0.004) less often than non-trainees. Residents also mainly used face to face to communicate risk to patients and used residents more often (46.6% vs. 2.7%, *p *< 0.0001) and pre-anesthesia clinic (42.2% vs. 57.3%, *p *= 0.02) and handouts/pamphlets (4.4% vs. 28.0%, *p *< 0.0001) less often compared to non-trainees. Residents communicated risk less often to referring providers (6.6% vs. 29.4%, *p *< 0.001).

**Table 2:** Duration of operative risk discussion, source of risk estimates, methods of communicating risk to patients, and communicating risk to referring provider

Questions	Total		Faculty/Affiliated Surgeons		Resident			
	(*n *= 120)		(*n *= 75)		(*n *= 45)			
	N(%)		N (%)		N (%)		*P* value*	
**When you discuss risk, about how much time do you spend?**								
Missing		1 (0.8)		0 (0)		1 (2.2)		**.004**
1-4 minutes		21 (17.5)		7 (9.3)		14 (31.1)		
5-9 minutes		54 (45.0)		33 (44.0)		21 (46.7)		
10-14 minutes		22 (18.3)		17 (22.7)		5 (11.1)		
> 14 minutes		22 (18.3)		18 (24.0)		4 (8.9)		
**Source of risk estimates**								
Online risk calculator								
Never (0%)	18 (15.0)		13 (17.3)		5 (11.1)		**.002**	
Rarely (1-24%)	37 (30.8)		25 (33.3)		12 (26.7)			
Sometimes (25-49%)	40 (33.3)		17 (22.7)		23 (51.1)			
Most of the time (50-74%)	13 (10.8)		8 (10.7)		5 (11.1)			
Always (75-100%)	12 (10.0)		12 (16.0)		0 (0)			
Prior experience								
Rarely (1-24%)	9 (7.5)		5 (6.7)		4 (8.9)		**<.0001**	
Sometimes (25-49%)	24 (20.0)		11 (14.7)		13 (28.9)			
Most of the time (50-74%)	41 (34.2)		19 (25.3)		22 (48.9)			
Always (75-100%)	46 (38.3)		40 (53.3)		6 (13.3)			
Assessment of current literature								
Never (0%)	6 (5.0)		5 (6.7)		1 (2.2)		**.004**	
Rarely (1-24%)	8 (6.7)		2 (2.7)		6 (13.3)			
Sometimes (25-49%)	34 (28.3)		16 (21.3)		18 (40.0)			
Most of the time (50-74%)	42 (35.0)		27 (36.0)		15 (33.3)			
Always (75-100%)	30 (25.0)		25 (33.3)		5 (11.1)			
Other sources								
Missing	11 (9.2)		0 (0)		11 (24.4)		**<.0001**	
Never (0%)	79 (65.8)		54 (72.0)		25 (55.6)			
Rarely (1-24%)	12 (10.0)		9 (12.0)		3 (6.7)			
Sometimes (25-49%)	7 (5.8)		3 (4.0)		4 (8.9)			
Most of the time (50-74%)	5 (4.2)		4 (5.3)		1 (2.2)			
Always (75-100%)	6 (5.0)		5 (6.7)		1 (2.2)			
**Methods of communicating risk to patients**								
Pre-anesthesia clinic								
Missing	1 (0.8)		0 (0)		1 (2.2)		**.02**	
Never (0%)	6 (5.0)		4 (5.3)		2 (4.4)			
Rarely (1-24%)	13 (10.8)		9 (12.0)		4 (8.9)			
Sometimes (25-49%)	38 (31.7)		19 (25.3)		19 (42.2)			
Most of the time (50-74%)	46 (38.3)		28 (37.3)		18 (40.0)			
Always (75-100%)	16 (13.3)		15 (20.0)		1 (2.2)			
Face to face communication								
Never	0 (0)		0 (0)		0 (0)		.43	
Rarely (1-24%)	3 (2.5)		1 (1.3)		2 (4.4)			
Sometimes (25-49%)	9 (7.5)		4 (5.3)		5 (11.1)			
Most of the time (50-74%)	25 (20.8)		16 (21.3)		9 (20.0)			
Always (75-100%)	83 (69.2)		54 (72.0)		29 (64.4)			
Phone call								
Never (0%)	40 (33.3)		23 (30.7)		17 (37.8)		.74	
Rarely (1-24%)	56 (46.7)		38 (50.7)		18 (40.0)			
Sometimes (25-49%)	19 (15.8)		11 (14.7)		8 (17.8)			
Most of the time (50-74%)	4 (3.3)		2 (2.7)		2 (4.4)			
Always (75-100%)	1 (0.8)		1 (1.3)		0 (0)			
Rely on resident to communicate risk								
Missing	1 (0.8)		0 (0)		1 (2.2)		**<.0001**	
Never (0%)	47 (39.2)		34 (45.3)		13 (28.9)			
Rarely (1-24%)	34 (28.3)		30 (40.0)		4 (8.9)			
Sometimes (25-49%)	15 (12.5)		9 (12.0)		6 (13.3)			
Most of the time (50-74%)	12 (10.0)		2 (2.7)		10 (22.2)			
Always (75-100%)	11 (9.2)		0 (0)		11 (24.4)			
Rely on advance practice provider								
Never (0%)	53 (44.2)		35 (46.7)		18 (40.0)		.61	
Rarely (1-24%)	36 (30.0)		23 (30.7)		13 (28.9)			
Sometimes (25-49%)	26 (21.7)		15 (20.0)		11 (24.4)			
Most of the time (50-74%)	5 (4.2)		2 (2.7)		3 (6.7)			
Always	0 (0)		0 (0)		0 (0)			
Handout/pamphlet								
Never (0%)	40 (33.3)		28 (37.3)		12 (26.7)		**<.0001**	
Rarely (1-24%)	34 (28.3)		11 (14.7)		23 (51.1)			
Sometimes (25-49%)	23 (19.2)		15 (20.0)		8 (17.8)			
Most of the time (50-74%)	14 (11.7)		12 (16.0)		2 (4.4)			
Always (75-100%)	9 (7.5)		9 (12.0)		0 (0)			
Class/video								
Never (0%)	86 (71.7)		58 (77.3)		28 (62.2)		.15	
Rarely (1-24%)	22 (18.3)		9 (12.0)		13 (28.9)			
Sometimes (25-49%)	7 (5.8)		4 (5.3)		3 (6.7)			
Most of the time (50-74%)	1 (0.8)		1 (1.33)		0 (0)			
Always (75-100%)	4 (3.3)		3 (4.0)		1 (2.2)			
**How often do you or your team communicate patient risk to their referring provider?**								
Missing	1 (0.8)		0 (0)		1 (2.2)		**<.001**	
Never (0%)	12 (10.0)		2 (2.7)		10 (22.2)			
Rarely (1-24%)	45 (37.5)		25 (33.3)		20 (44.4)			
Sometimes (25-49%)	37 (30.8)		26 (34.7)		11 (24.4)			
Most of the time (50-74%)	16 (13.3)		14 (18.7)		2 (4.4)			
Always (75-100%)	9 (7.5)		8 (10.7)		1 (2.2)			

*P values are from Fischer’s exact test

Table 3 presents factors related to when surgeons might be more likely to use risk assessment tools. Among all 120 surgeons, 90.0% were more likely to use risk assessment tools when patients had significant risk factors, 76.7% when patients were >65 years old, 70.0% to dissuade patients/families from surgery, 69.2% when patients asked about risks, and 49.2% when patients needed emergency operations. There were no statistically significant differences between faculty and affiliate surgeons and residents on these attitudes.

**Table 3:** Factors related to likelihood of using risk assessment tool

Question	Total	Faculty	Resident	
	(*n *= 120)	(*n *= 75)	(*n *= 45)	
	N(%)	N (%)	N (%)	*P* value*
Patient age > 65 years	92 (76.7)	56 (74.7)	36 (80.0)	.66
Patient asks about risk	83 (69.2)	48 (64.0)	35 (77.8)	.15
Patient has significant risk factors	108 (90.0)	67 (89.3)	41 (91.1)	.99
To dissuade patient/family from surgery	84 (70.0)	54 (72.0)	30 (66.7)	.54
Emergent operation	59 (49.2)	42 (56.0)	17 (37.8)	.06

*P values are from Fischer’s exact test

Table 4 presents respondents’ attitudes towards barriers in using risk calculators and the utility of certain features of risk calculators. Among all respondents, 65.9% thought time was a moderate or significant barrier, 61.7% not being integrated into the electronic health record (EHR) or inaccessibility during patient visit, 59.2% trust in accuracy, 46.7% inability of patient to understand, and 32.5% patient language barrier. Compared to faculty and affiliate surgeons, residents thought that inaccessibility of the tool during the patient visit and native language of patient were more moderate or significant barriers. Among all 120 surgeons, 93.3% thought prediction of major complications and mortality made risk calculators very or extremely useful, 80.9% automatic integration of risk factors from the EHR, 80.0% prediction of post-surgery infection, 77.5% automatic recording of results into the EHR, 75.0% prediction of a good surgical outcome, and 48.3% prediction of minor complications. There were no statistically significant differences in attitudes toward features that make risk calculators useful between the faculty and residents.

**Table 4:** Potential barriers to use of risk assessment tool and features that make risk assessment tool useful

Question	Total	Faculty	Resident	
	(*n *= 120)	(*n *= 75)	(*n *= 45)	
	N(%)	N (%)	N (%)	*P* value*
**Potential barriers to use of risk calculator**				
The amount of time it takes to use				
Not a barrier	8 (6.7)	7 (9.3)	1 (2.2)	.08
A small barrier	33 (27.5)	25 (33.3)	8 (17.8)	
A moderate barrier	47 (39.2)	25 (33.3)	22 (48.9)	
A significant barrier	32 (26.7)	18 (24.0)	14 (31.1)	
The risk calculator not being integrated with the EHR				
Not a barrier	13 (10.8)	12 (16.0)	1 (2.2)	.08
A small barrier	33 (27.5)	21 (28.0)	12 (26.7)	
A moderate barrier	39 (32.5)	21 (28.0)	18 (40.0)	
A significant barrier	35 (29.2)	21 (28.0)	14 (31.1)	
The inability of patients to understand the results				
Not a barrier	16 (13.3)	11 (14.7)	5 (11.1)	.96
A small barrier	48 (40.0)	29 (38.7)	19 (42.2)	
A moderate barrier	32 (26.7)	20 (16.7)	12 (26.7)	
A significant barrier	24 (20.0)	15 (20.0)	9 (20.0)	
The inaccessibility of risk tool during patient visit				
Missing	1 (0.8)	0 (0)	1 (2.2)	**.02**
Not a barrier	13 (10.8)	12 (16.0)	1 (2.2)	
A small barrier	32 (26.7)	21 (28.0)	11 (24.4)	
A moderate barrier	42 (35.0)	20 (26.7)	22 (48.9)	
A significant barrier	32 (26.7)	22 (29.3)	10 (22.2)	
Trust of the risk tool accuracy				
Not a barrier	17 (14.2)	12 (16.0)	5 (11.1)	.16
A small barrier	32 (26.7)	19 (25.3)	13 (28.9)	
A moderate barrier	44 (36.7)	23 (30.7)	21 (46.7)	
A significant barrier	27 (22.5)	21 (28.0)	6 (13.3)	
Native language of patient				
Not a barrier	32 (26.7)	26 (34.7)	6 (13.3)	**.006**
A small barrier	49 (40.8)	29 (38.7)	20 (44.4)	
A moderate barrier	26 (21.7)	10 (13.3)	16 (35.6)	
A significant barrier	13 (10.8)	10 (13.3)	3 (6.7)	
**Potential features that make risk calculator useful**				
Prediction of good surgical outcome				
Not useful at all	3 (2.5)	2 (2.7)	1 (2.2)	.53
Somewhat useful	23 (19.2)	16 (21.3)	7 (15.6)	
Very useful	41 (34.2)	28 (37.3)	13 (28.9)	
Extremely useful	49 (40.8)	26 (34.7)	23 (51.1)	
Don’t know	4 (3.3)	3 (4.0)	1 (2.2)	
Prediction of minor complications				
Not useful at all	9 (7.5)	6 (8.0)	3 (6.7)	.12
Somewhat useful	51 (42.5)	37 (49.3)	14 (31.1)	
Very useful	36 (30.0)	22 (29.3)	14 (31.1)	
Extremely useful	22 (18.3)	9 (12.0)	13 (28.9)	
Don’t know	2 (1.7)	1 (1.3)	1 (2.2)	
Prediction of major complications				
Not useful at all	1 (0.8)	1 (1.3)	0 (0)	.95
Somewhat useful	5 (4.2)	4 (5.3)	1 (2.2)	
Very useful	34 (28.3)	21 (28.0)	13 (28.9)	
Extremely useful	78 (65.0)	48 (64.0)	30 (66.7)	
Don’t know	2 (1.7)	1 (1.3)	1 (2.2)	
Prediction of mortality				
Not useful at all	1 (0.8)	1 (1.3)	0 (0)	.57
Somewhat useful	6 (5.0)	5 (6.7)	1 (2.2)	
Very useful	27 (22.5)	16 (21.3)	11 (24.4)	
Extremely useful	85 (70.8)	53 (70.7)	32 (71.1)	
Don’t know	1 (0.8)	0 (0)	1 (2.2)	
Automatic integration of risk factors from the EHR				
Not useful at all	4 (3.3)	4 (5.3)	0 (0)	.16
Somewhat useful	18 (15.0)	14 (18.7)	4 (8.9)	
Very useful	26 (21.7)	16 (21.3)	10 (22.2)	
Extremely useful	71 (59.2)	41 (54.7)	30 (66.7)	
Don’t know	1 (0.8)	0 (0)	1 (2.2)	
Automatic recording of results into the EHR				
Not useful at all	5 (4.2)	5 (6.7)	0 (0)	.20
Somewhat useful	18 (15.0)	13 (17.3)	5 (11.1)	
Very useful	30 (25.0)	20 (26.7)	10 (22.2)	
Extremely useful	63 (52.5)	34 (45.3)	29 (64.4)	
Don’t know	4 (3.3)	3 (4.0)	1 (2.2)	
Prediction of post-surgery infection				
Not useful at all	2 (1.7)	2 (2.7)	0 (0)	.11
Somewhat useful	21 (17.5)	17 (22.7)	4 (8.9)	
Very useful	49 (40.8)	30 (40.0)	19 (42.2)	
Extremely useful	47 (39.2)	26 (34.7)	21 (46.7)	
Don’t know	1 (0.8)	0 (0)	1 (2.2)	

*Abbreviation:* *EHR* electronic health record

*P values are from Fischer’s exact test

We also examined surgeon attitudes toward risk discussions with patients and use of risk assessment tools between the 21 affiliate and the 54 faculty surgeons. The attitudes were similar for all questions, with the exceptions of use of residents to communicate risk and whether they were more likely to use risk assessment tools for emergent operations. Faculty used residents more to communicate risk (70% for faculty vs. 14% for affiliate surgeons rarely to always, *p *< 0.0001) and they were more likely to use risk assessment tools for emergent operations (63% vs. 38%, *p *= 0.05). We also examined surgeon characteristics (gender, age, years of practice, education, type and bed size of facility practicing in) related to whether they used risk assessment tools never/rarely (*n *= 55), sometimes (*n *= 40), or most of the time/always (*n *= 25) (data not presented). We found no consistent trends between any of the surgeon characteristics and their use of risk assessment tools.

## Discussion

This study found that a small percentage of surgeons (20.8%) used on-line risk calculators most or all of the time; that attendings were more likely to use prior experience and current literature and residents more likely to use risk calculators as a source of risk estimates; and that time was the number one barrier to use of risk calculators. The surgeons were also more likely to use risk calculators when patients were older, had significant risk factors, to dissuade patients and families about surgery when risk was high, or when patients asked about risk. Risk calculators were thought to be more useful for prediction of major complications, mortality, and post-surgical infection; when they were built into the EHR, when they automatically recorded results into the EHR; and when they predicted a good surgical outcome; they were less useful for minor complications. Top barriers for use of risk calculators, in addition to time, were thought to be accessibility and not being in the EHR, trust, inability of patients to understand, and language barriers.

It is not surprising that residents would rely more on risk calculators and less on their prior experience in estimating preoperative risk. The average age of the residents was 30.7 years, so they might be more in tune with computer technology, and they had more limited prior clinical experience. The fact that the surgeons were more interested in risk prediction tools for older patients with more risk factors and to dissuade patients with very high risk agrees with our previous research which showed that surgeons thought a risk calculator was more useful for high-risk patients and less useful for low-risk patients [2, 4].

Meguid, et al, [14–18] have developed a surgical risk calculator that has some of the features thought to make a risk calculator useful and to avoid some of the perceived barriers. Called the Surgical Risk Preoperative Assessment System (SURPAS), the risk calculator covers 9 surgical specialties (general, gynecology, neurosurgery, orthopedics, otolaryngology, plastics, thoracic, urology, and vascular), only requires the input of 8 preoperative variables (4 related to the operation—CPT-specific event rate, work RVU, inpatient/outpatient, and specialty; and 4 related to the patient—age, ASA class, functional health status, and emergency), and calculates a patient’s risk for 12 postoperative adverse events (30-day mortality, overall morbidity, unplanned readmission, discharge not to home, and the 8 specific complications of infection, cardiac, pulmonary, renal, UTI, VTE, bleeding, and stroke). Thus, the calculator is easy to use, taking only a few minutes to input data (time barrier); covers the major postoperative complications; is built into the EHR at our local health system and can be integrated into the EHR at other institutions; and writes a note into the patient’s EHR chart. Further, multiple validation studies have been done using internal [17–21] and external [22–25] validation methods, to address the issue of “trust” in the accuracy of the models. Several pilot studies have also been completed recently showing that after SURPAS patients understand their risks for surgery very well [2] and have improved satisfaction, comfort, and anxiety about their operations [10]. If surgeons at other institutions wish to use SURPAS, they can either use an on-line version at https://surpass.agilemd.com, or if they want SURPAS to be built into their own local EHR, they can access the SURPAS statistical models at https://github.com/katiecolborn/SURPAS.

Other studies have shown that risk assessment tools are underutilized by trainees, favoring traditional models of communication due to lack of an electronic health record and clinical workflow interruption [26]. This remains true for attending surgeons as well. A patient’s native language may also pose a barrier to using risk calculator tools in hospitals where interpreter services are not readily available. A formal handout given at the end of the visit, complete with visuals of risk, may help reinforce the preoperative risk discussion. When surgeons forego using a surgical risk calculator, they misestimate a patient’s risk of postoperative adverse events regardless of level of training [11].

In order to successfully implement surgical risk tools, especially in a high-volume clinic setting, accessing and using these tools must be quick, seamless, and accurate. Both ACS-NSQIP and VASQIP require manual entry of over 20 pre-operative variables, including laboratory values that may not be readily available for all pre-operative patients. These factors might preclude widespread use in a large volume pre-operative clinic setting where surgeons may only have 15 minutes allotted for the entire patient encounter.

This study had two major limitations: (1) Respondents were from just one health system (UCHealth). However, the health system did have different types of hospitals, including a large university hospital, several community hospitals, and a few rural hospitals; and (2) The overall response rate was only 46.0%. However, this was somewhat better than the typical response rates of 20-30% observed in other physician surveys [27].

## Conclusions

Only twenty percent of the surveyed surgeons used a risk calculator most or all of the time. Attending surgeons were more likely to use prior experience and current literature as sources for risk information, while residents were more likely to use risk calculators. Time, accessibility, and trust in accuracy were cited as the most important barriers to use of a risk calculator.

## Supplementary Information

**Additional file 1: Fig. S1. **The 16-question survey distributed to surgeons to assess attitudes about surgical risk assessment.

## Data Availability

The datasets used and/or analyzed during the current study are available from the corresponding author on reasonable request.

## Abbreviations

COMIRB: Colorado Multiple Institutional Review Board
REDCap: Research Electronic Data Capture
ACS-NSQIP: American College of Surgeons’ Surgical Risk Calculator
VASQIP: Veterans Affairs Surgical Quality Improvement Program
UCH: University of Colorado Hospital
SURPAS: Surgical Risk Preoperative Assessment System
