# Supplementary material for: Attitudes about use of preoperative risk assessment tools: a survey of surgeons and surgical residents in an academic health system
Source: Patient Saf Surg. 2022 Mar 17;16:13. doi: 10.1186/s13037-022-00320-1 (PMC8932286; doi:10.1186/s13037-022-00320-1)
Supplement: Supplementary file 1 — Additional file 1: Fig. S1. The 16-question survey distributed to surgeons to assess attitudes about surgical risk assessment. [file 13037_2022_320_MOESM1_ESM.pdf]

Appendix 1. Survey Emailed to Surgeons.

Understanding Attitudes Towards and Techniques of Surgical Preoperative Risk Assessment

The results of this survey will be used in the development of a computer-based risk assessment tool for patients considering surgical options.

1. What is your surgical specialty?

- Cardiac
- General
- Gynecologic
- Neurological
- Orthopedic
- Other (please specify): ___
- Otolaryngologic
- Plastic
- Thoracic
- Urologic
- Vascular

2. How many years have you been in practice after completing post-graduate clinical training? _____

3 What is your gender?

- Male
- Female
- Other
- I prefer not to answer

4. What is your age? ______

5. What graduate or doctoral degrees have you obtained? (please check all that apply)

- MD
- DO
- DNP
- PhD
- MBA
- MPH
- MS
- MSN
- PA-C
- Other (please specify): ___

6. Which option best describes your primary workplace?

- Academic Hospital
- Urban Community Hospital
- Rural Hospital

- Other (please specify): ___

7. What is the bed size of your hospital?

- 0-199
- 200-399
- 400-599 (Denver Health)
- 600-799 (University of Colorado Hospital)
- 800+

8. How often do you discuss the operative risk with your patient preoperatively?

- Never (0%)
- Rarely (1-24%)
- Sometimes (25-49%)
- Most of the time (50-74%)
- Always (75-100%)

9. How often do you use each of the following methods of risk assessment & communication? (Please choose one for each method)

	Never (0%)	Rarely (1-24%)	Sometimes (25-49%)	Most of the time (50-74%)	Always (75-100%)
Pre-anesthesia clinic (e.g. Pre-procedure services)	○	○	○	○	○
Face to face communication	○	○	○	○	○
Phone call	○	○	○	○	○
Rely on resident to communicate risk	○	○	○	○	○
Rely on Advanced Practice Provider (NP, PA) to communicate risk	○	○	○	○	○
Handout/Pamphlet	○	○	○	○	○
Class/Video	○	○	○	○	○

10. When you discuss risk with a patient, about how much time do you usually spend?

- I do not spend any time
- 1-4 minutes
- 5-9 minutes
- 10-14 minutes
- >15 minutes

11. How often do you or your team communicate patient preoperative risk information to the patient's referring provider or primary care provider?

- Never (0%)
- Rarely (1-24%)
- Sometimes (25-49%)

- Most of the time (50-74%)
- Always (75-100%)

12. When you are evaluating a patient for surgery, what do you use for your source of risk information?

	Never (0%)	Rarely (1-24%)	Sometimes (25-49%)	Most of the time (50-74%)	Always (75-100%)
Online risk calculator (e.g. ACS Surgical Risk Calculator)	○	○	○	○	○
Prior experience	○	○	○	○	○
Assessment of current literature	○	○	○	○	○
Other	○	○	○	○	○

12b. If you chose "Other" please specify the risk assessment tool(s) you utilize. _____________

13. When are you more likely to use a risk assessment tool?

	No	Yes
Patient age >65	○	○
Patient asks about risk	○	○
Patient has significant risk factors	○	○
To dissuade patient/family from surgery	○	○
Emergent operation	○	○

14. What operative risk assessment tool features would be useful to your surgical team in a risk assessment tool?

	Not useful at all	Somewhat useful	Very useful	Extremely useful	Don’t know
Prediction of good surgical outcome	○	○	○	○	○
Prediction of minor complications	○	○	○	○	○
Prediction of major complications	○	○	○	○	○
Prediction of mortality	○	○	○	○	○
Automatic integration of risk factors from EHR	○	○	○	○	○
Automatic recording of results into the EHR	○	○	○	○	○
Prediction of post-surgery infection	○	○	○	○	○

15. How much of a barrier is each of the following factors to using a formal risk assessment tool?

	Not a barrier	A small barrier	A moderate barrier	A significant barrier
The amount of time it takes to use	○	○	○	○

The inability of the risk calculator to integrate with the EHR	○	○	○	○
The inability of patients to understand the results	○	○	○	○
The inaccessibility of risk assessment tool during patient visits	○	○	○	○
Trust of risk tool accuracy	○	○	○	○
Native language of patient	○	○	○	○

16. To be entered into a raffle for a $50 Amazon gift card, please enter your email address. Incomplete surveys will not be considered in the raffle. ___________________

Thank you for your participation in completing this survey. We greatly appreciate your time and help with this project.
